# Mechanisms of sex determination and transmission ratio distortion in *Aedes aegypti*

**DOI:** 10.1186/s13071-016-1331-x

**Published:** 2016-01-28

**Authors:** Kim Phuc Hoang, Tze Min Teo, Thien Xuan Ho, Vinh Sy Le

**Affiliations:** University of Engineering and Technology, Vietnam National University, Hanoi, 144 Xuan Thuy, Cau Giay, 10000 Hanoi Vietnam; Advanced Agriecological Research Sdn. Bhd, No. 11 Jalan Teknologi 3/6, 47810 Petaling Jaya, Selangor Malaysia; Department of Plant Pathology, University of Arkansas, 495 N Campus Drive, Fayetteville, AR 72701 USA

**Keywords:** Aedes aegypti, Sex determination, Transformer-2, Meiotic drive, Spermatogenesis, Culicinae

## Abstract

**Background:**

More effective mosquito control strategies are urgently required due to the increasing prevalence of insecticide resistance. The sterile insect technique (SIT) and the release of insects carrying a dominant lethal allele (RIDL) are two proposed methods for environmentally-friendly, species-targeted population control. These methods may be more suitable for developing countries if producers reduce the cost of rearing insects. The cost of control programs could be reduced by producing all-male mosquito populations to circumvent the isolation of females before release without reducing male mating competitiveness caused by transgenes.

**Results:**

An RNAi construct targeting the RNA recognition motif of the *Aedes aegypti transformer-2* (*tra-2*) gene does not trigger female-to-male sex conversion as commonly observed among dipterous insects. Instead, homozygous insects show greater mortality among m-chromosome-bearing sperm and mm zygotes, yielding up to 100 % males in the subsequent generations. The performance of transgenic males was not significantly different to wild-type males in narrow-cage competitive mating experiments.

**Conclusion:**

Our data provide preliminary evidence that the knockdown of *Ae. aegypti tra-2* gene expression causes segregation distortion acting at the level of gametic function, which is reinforced by sex-specific zygotic lethality. This finding could promote the development of new synthetic sex distorter systems for the production of genetic sexing mosquito strains.

**Electronic supplementary material:**

The online version of this article (doi:10.1186/s13071-016-1331-x) contains supplementary material, which is available to authorized users.

## Background

The global economic burden of mosquito-borne diseases continues to grow due to the failure of current control measures. Two novel methods based on the suppression of reproduction have been applied in mosquitoes, namely the sterile insect technique (SIT) and the release of insects carrying a dominant lethal allele (RIDL) [1–5]. However, these methods produce offspring of both sexes and 0.5–1 % of mosquitoes are physically misidentified as males during the sexing step in these techniques. In mosquitoes, it is an essential prerequisite to release only males because the females are blood feeders and disease vectors [6]. Genetic sexing strains based on the RIDL system have been developed to produce flightless females [7] but the trait also affects males, reducing their time in flight by 21 % compared to wild-type mosquitoes [8]. These drawbacks have led to research focusing on the exploitation of sex and sex-ratio determination mechanisms to control mosquito populations.

A recent publication [9] has shown that a new autosomal sex-determining allele, which may or may not exhibit all the other characteristics of a sex chromosome, succeeds when linked to a sex-specific meiotic driver (*MD*). The new sex-determining allele benefits from confining the driving allele to the sex in which it gains the benefit of drive. *Aedes aegypti* carrying a distorter gene *D* shows meiotic drive when associated with the male-determining *M* gene on the M-chromosome (chromosome I). Although the molecular basis of the *MD* system is unclear, it causes a highly male-biased sex ratio phenotype in *Ae. aegypti* and all *MD* males are genetic males. The excess of *MD* males involves isochromatid breakage in the m-chromosome before or during the diplotene phase of the first meiotic division, although some m-sperm dysfunctions manifest later in spermatogenesis after the segregation of homologous chromosomes [10–13]. Intriguingly, *mD* was not resistant to *MD* as proposed, and the *MD-T1* enhancer was identified as a translocation from chromosome III [10], indicating that genetic dominance is not responsible for *MD* in *Ae. aegypti*. The molecular basis of *MD* and transmission ratio distortion in *Drosophila simulans* has recently been shown to depend on endogenous retrotransposon-dependent RNA interference (RNAi) [14, 15]. In a recent publication, the ectopic expression of an M-locus (*Nix* gene) in *Ae. aegypti* female embryos caused the initial development of male genitals and testes in more than 69 % (16/23) of genetic females [16]. *Nix* knockout with CRISPR/Cas9 generated largely feminized genetic males. The authors concluded that *Nix* is the male-determining *M* gene of *Ae. aegypti* which is both required and sufficient to initiate male development. The results indicated that more genes may be required to complete male development, and this may complicate any transgenic strategy by affecting the fitness of transgenic insects. The detailed description of feminized antennae in *nix*^–^ males and the failure of these feminized males to obtain a blood-meal indicated that the *M* gene does not affect the development of a male proboscis. However, the mandibles and maxillae of the biting fascicle in the *nix*^+^ females were not described. Female-to-male sex conversion mosquitoes are not harmless if the biting fascicle is still present and biting insects would therefore be unsuitable for a control program.

The *D. melanogaster transformer-2* (*tra-2*) gene plays essential roles in both sex determination in the female soma and spermatogenesis in the male germ cells. The *tra-2* gene encodes a non-sex-specific auxiliary splicing factor that promotes female sexual differentiation in insects by interacting with the female-specific product of the *transformer* (*tra*) gene [17–20]. The TRA/TRA-2 protein complex then regulates other genes that control sexual differentiation, including *doublesex* (*dsx*) [21, 22]. In *D. melanogaster*, *tra-2* is also involved in the specification of the male germ line, and a null *tra-2* allele causes male sterility although the underlying mechanism is unclear [23–26]. The secondary structure of the TRA-2 protein comprises anarginine/serine-rich (RS) domain, an 81-residue RNA recognition motif (RRM), a 19-residue linker region unique to TRA-2 proteins, and a second C-terminal RS domain. The RRM and linker region are the most conserved components of TRA-2 proteins among dipteran and non-dipteran insects [27, 28]. The injection of *Drosophila*, *Ceratitis* and *Anastrepha* spp. embryos with synthetic *tra-2* dsRNA causes the degradation of *tra-2* mRNA and the conversion of females into pseudomales [17–21]. The *Ae.aegypti* genome contains four putative *tra-2* homologs, namely *AAEL009224*, *AAEL006416*, *AAEL009222* and *AAEL004293* [27, 29] [VectorBase: *AAEL009224*, *AAEL006416*, *AAEL009222, AAEL004293*]. The direct injection of synthetic dsRNAs representing three of these genes into *Ae. aegypti* embryos did not affect *dsx* splicing during the larval stages, and did not produce intersex adult phenotypes [27].

Six transgenic *Ae. aegypti* Rock lines were therefore produced expressing the *tra-2* RNAi construct Zoo-2 [30], which targets the *tra-2* RRM (*AAEL004293-*RRM) responsible for protein–protein interactions and the splicing of *dsx* mRNA. The RNAi construct was driven by the *tet* operator (*tet*O) from the *tet* transactivator system (*t*TA), which is generally active but can be suppressed in crosses with *t*TA transgenic mosquitoes when tetracycline analogs are also supplied (Additional file 1: Figure S1) [31, 32]. The *tra-2* RNAi construct also features a *DsRed-2* marker gene linked to the RNAi sequence, which allows transgenic and wild-type flies to be distinguished in genetic crosses. Teo [30] tested the interaction between the Tet-responsive control element in line 6 and the Tet-off transactivator protein from another transgenic line. Homozygous *tra-2* RNAi females (line 6) were thus crossed with homozygous *LA513* males (Additional file 2: Table S1) [33] carrying the *t*TA transactivator gene, but homozygous females + Tet yielded progeny with a 1:1 sex ratio and normal hatching and survival rates, whereas homozygous females –Tet yielded progeny showed a significant bias towards male without intersex adult phenotypes, indicating that the abnormal sex ratio should be attributed to the *t*TA protein of *LA513* fathers. The *LA513* construct was also designed to kill the carrier’s offspring from the third larval stage, hence also reducing the survival in the absence of Tet. Quantitative real-time PCR was used to measure the level of *tra-2* mRNA remaining after knockdown in homozygous line 6 and wild-type Rock males, showing that the *tra-2* expression level was reduced by an average 48.7 % in line 6 relative to wild-type insects (S*tandard deviation = 4.98*) (Additional file 3: Figure S2) [30]. Based on evidence from other dipterous species, Teo considered the possibility of female-to-male sex conversion, but this would imply that up to 50 % of the male progeny carry mm chromosomes and solid evidence based on karyotyping, sex-diagnostic PCR or crossing analysis were not shown [30].

To investigate the bias towards male progeny and the molecular basis of *Ae. Aegypti* sex determination by *tra-2*, we carried out a phylogenetic analysis of the RRM regions of putative TRA-2 homologs in Culicinae mosquitoes followed by a more detailed genetic analysis of line 6 [30]. We tested the mating capacity of transgenic males in competition experiments. We also injected synthetic *tra-2* RNAi constructs into *Ae. aegypti* embryos to observe their effect during transient expression*.* Here we report a potentially novel mosquito sex-determination mechanism in which the *tra-2* gene is responsible for segregation distortion (*SD*) and female lethality in the subsequent generation of mosquitoes.

## Methods

### Phylogenetic analysis

The RRM amino acid sequences described herein are *D. melanogastertra-2* isoform C, E [GenBank:*NP 476766.1*], *M. domesticatra-2* [GenBank:*XP 005185276.1*], *An. gambiaetra-2* [VectorBase:*AGAP006798*] and*Cx. quinquesfasciatustra-2* [VectorBase:*CPIJ016646*]. Three further *Ae. aegyptitra-2* RRMs (*AAAEL009222, AAAEL006416* and *AAAEL009224* [VectorBase]) were also analyzed, as well as *Ae. aegypti*, *Ae. albopictus* and *Ae. polynesiensis* RRM1 [GenBank: *KJ147318, KJ147321, KJ147316* and *KJ147314*] and RRM2 [GenBank: *KJ147317, KJ147320* and *KJ147315*]. RRM1 is the RRM domain of *AAEL004293.* The evolutionary history was inferred using the maximum likelihood method based on the JTT matrix model. The bootstrap consensus tree inferred from 1000 replicates was taken to represent the evolutionary history of the taxa. Branches corresponding to partitions reproduced in less than 50 % of the bootstrap replicates were collapsed. The percentage of replicate trees in which the associated taxa clustered together in the bootstrap test (1000 replicates) is shown next to the branches. Initial trees for the heuristic search were created by applying the neighbor-joining method to a matrix of pairwise distances estimated using a JTT model. Nine amino acid sequences were analyzed. Positions containing gaps and missing data were eliminated. The final dataset contained 78 positions. Evolutionary analysis was carried out using MEGA5 [34, 35].

### Plasmid construction

The Zoo-2 construct and line 6 were prepared as previously described [30]. Briefly, the *Ae. aegypti AAEL004293* sequence, which shows the greatest similarity to *D. melanogastertra-2*, was screened to find the RRM (predicted by PROSITE), and a 135-bp sequence was used to create the RNAi construct. This was assembled using a *PiggyBac* backbone from the *LA513* plasmid [33] fused to a PCR fragment containing a *tet* operator sequence and minimal promoter (*tet*O7-*CMV*) and two *Ae. aegypti tra-2* inverted repeats connected by an intron. The *LA513* construct was digested with SrfI and AscI to remove the *t*TA/*tet*O7 transactivator cassette and the backbone was trimmed to blunt ends before re-ligation with T4 DNA polymerase.

A 1770-bp DNA fragment containing (1) 376 bp of the *tet*O7-*CMV* cassette [1409–1784, GenBank:KC710231.1]; (2) 285 bp of the *D. melanogaster NIPP1* gene [512–793, GenBank:AJ427611.1]; and (3) 922 bp of the rabbit β-globulin 3′ untranslated region [14515–15435, GenBank:AB236435.1] was amplified from an available DNA template using the primers NotIF (5′-CGA TCA GCG GCC GCA AAG CTG GGA GCA ATT CTC GCA-3′) and NaeIR (5′-GCA GTC GCC GGC GCT ATG GGC ATA TGT TGC CA-3′). The sequences were amplified by PCR in a 25-μl reaction comprising 2.5 μl PCR buffer, 1.5 μl 25 mM MgCl_2_, 0.5 μl each dNTP (10 mM) and 0.5 μl of each primer (10 pmol/μl). This was supplemented with 0.15 μl *Taq* DNA polymerase (5 U/μl) and 10–40 ng of template DNA. After heating to 94 °C for 4 min, we carried out 35 cycles of denaturation at 94 °C for 30 s, annealing at 54 °C for 90 s and extension at 72 °C for 150 s before holding at 72 °C for 10 min. The re-ligated *LA513* was mixed with the PCR fragment at a 3:1 molar ratio and digested with NotI and NaeI. The resulting plasmid was cloned in competent cells then isolated and digested with SacII and BamHI to remove the *NIPP1* gene, leaving the *tet*O7-*CMV* minimal promoter cassette and rabbit β-globulin 3′-UTR available for the *tra-2* RNAi core sequence. The *tra-2* sequence was amplified using four different primers in three different combinations. All primer sequences used to amplify the RRM sequence and the *tra-2* intron fragment are listed in Additional file 2: Table S8. Primers 1, 2, 3 and 4 were flanked by SacII, XhoI, BamHI and EcoRI sites, respectively.

The inverted repeats of the Zoo-2 construct were connected by a 245-bp *tra-2* intron fragment (1220424–1220669 in supercont1.113). The PCR products amplified by primer pairs 1/2 and 3/4 were digested with XhoI and EcoRI and then ligated to the *tra-2* intron fragment, which was amplified using primers 5 and 6. The RNAi core structures were then digested with SacII and BamHI and mixed with the linear plasmid described above in a 1:3 molar ratio. The size of the final plasmid was 11,448 bp.

### Mosquito transformation and analysis

*Ae. aegypti* Rock mosquitoes (*Ae. aegypti* Rockefeller line, Liverpool School of Tropical Medicine) and transgenic line 6 were reared using a standard protocol [36]. The posterior poles of 2500 Rock embryos were microinjected with a cocktail of the Zoo-2 and transposase constructs (0.6 and 0.3 μg/μl, respectively) using a Femtotip II with the NiKon Eclipse TS100 microinjector system. The transposase construct was a helper plasmid carrying the *D. melanogaster* heat shock 70 (hsp70) promoter driving the expression of the *PiggyBac* transposase. Six transformed lines were established, distinguished by remarkable differences in *DsRed-2* marker gene expression in G1 larvae, and we used line 6 [30] for the further experiments described herein.

Inverse PCR was used to identify genomic sequences adjacent to the insertion site of the *tra-2* RNAi construct as previously described [37]. The *PiggyBac* 5′ sequence was used to design two pairs of nested primers which amplify the same insertion and flanking regions but generate products differing in size by 214 bp (TaqαI-PB5-1, 5′-GAT GAG GTA CAT GAA GTG CAG CCA-3′ and TaqαI-PB5-2, 5′-CAT GCG TCA TTT TGA CTC ACG C-3′; TaqαI-PB5-3, 5′-TAG CCG AGT CTC TGC ACT GAA C-3′ and TaqαI-PB5-4, 5′-CAG TGA CAC TTA CCG CAT TGA C-3′). The sequences were amplified by PCR in a 25-μl reaction with the same ingredients listed above except we used 10–20 ng of template DNA. After heating to 94 °C for 4 min, we carried out 35 cycles of denaturation at 94 °C for 45 s, annealing at 55 °C for 60 s and extension at 72 °C for 60 s before holding at 72 °C for 10 min. The first primer pair (TaqαI-PB5-1 and TaqαI-PB5-2) yielded a 981-bp product whereas the second primer pair (TaqαI-PB5-3 and TaqαI-PB5-4) yielded a 767-bp product. The nested primer binding sites allowed us to verify that the amplification was reproducible and also confirmed the single insertion site. If there was more than one insertion, each PCR should produce more than one product after 35 cycles due to the presence of identical primer binding sites at each locus.

The flanking regions were sequenced and two diagnostic primers were designed to test the allelic status of the insertion (Flk-2, 5′-GCA GAA GCT TGA ATT GAA GCC TCT GA-3′, and PB5-2, 5′-GCA GAG AGG ATA TGC TCA TCG TCT-3′). Wild-type insects yielded a 275-bp product whereas heterozygotes yielded this plus an additional 584-bp product containing the insert. Mass crossing between heterozygous males and females allowed the selection of second-instar larvae and pupae in the next generation showing the brightest DsRed fluorescence, corresponding to homozygous insertions. This was confirmed by diagnostic PCR once the individuals had reached reproductive age. Eggs from homozygous parents were pooled to establish the homozygous line. Larvae and pupae with a *DsRed-2* marker were screened using an Olympus SZX9 fluorescence stereo microscope. After separating male and female pupae, the number of individuals expressing the *DsRed-2* marker was counted in each group.

To prepare mosquitoes for genetic crosses, each half-gravid female from the homozygous line was separately collected in a *Drosophila* vial containing a wet filter paper disk and allowed to lay eggs in dark. Each egg batch was hatched in deoxygenated water and grown separately. When the progeny developed to the pupal stage, isofemale siblings were placed into two cages (20 cm x 20 cm) for males and females, every day until the last pupae eclosed, and the sex ratio was immediately counted. This process usually took 4–6 days at 27 ± 2 °C, depending on the size of each family. Mosquitoes were fed with 20 % sugar or honey solution and were kept for 3 additional days to ensure that the last eclosed insect was 3 days old. The transgenic mosquitoes used in the crosses varied from 3 to 9 days old. This procedure was used for mosquitoes in the experiments in Fig. 2 (a, b and c) and Fig. 3.

### Quantitative RT-PCR (qPCR)

The expression of *tra-2* in transgenic males and wild-type Rock males was measured by qPCR [30]. Briefly, male pupae were collected in pools of five male pupae in three replicates for RNA extraction. Template cDNA was obtained from one μg DNase treated RNA of each sample, using SuperScript VILO cDNA Synthesis Kit (Invitrogen) for transcription and then performed using 1 to 20 dilutions of the cDNAs using SYBR Green I for qPCR. Three qPCR assays were carried out to ensure the results were consistent and an average expression level was calculated. After heating to 95C for 15 min, we carried out 45 cycles of denaturation for 10 s at 95 °C, annealing for 10 s and extension for 20 s at 72 °C. The annealing temperature was primer-specific, i.e. 54 °C for the *tra-2* gene (F 5′-GAA ACA TCG GCC TGT TCA TC-3′ and R 5′-GCT GGT TTC GTT GGT GTA GC-3′), and 59 °C for the housekeeping gene *Actin5C* (F 5′-ATC GTA CGA ACT TCC CGA TG-3′ and R 5′-ACA GAT CCT TTC GGA TGT CG-3′). The *tra-2* primers generated a 120-bp product from *tra-2* exon 1 55 bp upstream of the RNAi target site, whereas the *Actin5C* primers generated a 125-bp fragment. Readings were taken every 1 °C with a 1-s hold for the melting curve between 50 °C and 95 °C. The relative standard curve method was used to calculate expression levels (ABI Prism 7700 Manual, Applied Biosystems) with *Actin5C* as an endogenous reference and the wild-type Rock mRNA as an exogenous calibrator.

### RRM sequences from other Culicinae mosquitoes and microinjection of *Ae. aegypti* with the *tra-2* RNAi construct

*Ae. albopictus* mosquitoes were collected from Northern Vietnam, Central Laos (Khamcot) and Malaysia (Kuala Lumpur). Larval morphology was confirmed and DNA was extracted from five individuals from each site using the Qiagen DNA extraction kit, for RRM amplification and sequencing. Seven dried *Ae. polynesiensis* specimens were obtained from Fiji and two from Tahiti. The morphology of these samples was confirmed and DNA extracted and tested as above. RRM regions from *Ae. albopictus* and *Ae. polynesiensis* were amplified by PCR using primers CLF (5′-AGT AAG TGC CTC GGT GTG TTC GGC CT-3′) and CLR (5′-CCG GTC ACC GAA TAA TCC ACT CAA-3′) spanning a 26-bp proximal segment and a 23-bp distal segment of the *Ae. aegypti* supercontig 1.113 (1221182–1220943). The sequences were amplified by PCR in a 25-μl reaction comprising 2.5 μl PCR buffer, 1.5 μl 25 mM MgCl_2_, 0.5 μl each dNTP (10 mM) and 0.5 μl of each primer (10 pmol/μl) supplemented with 0.15 μl *Taq* DNA polymerase (5 U/μl) and 10–40 ng of template DNA. After heating to 94 °C for 4 min, we carried out three amplification cycles at 94 °C for 30 s, 59 °C for 30 s and 72 °C for 45 s, three cycles at 94 °C for 30 s, 57 °C for 30 s and 72 °C for 45 s, and 35 cycles at 94 °C for 30 s, 54 °C for 30 s and 72 °C for 45 s, before holding at 72 °C for 10 min. The 240-bp product among multiple bands was purified from agarose gels using the Qiagen extraction kit. Strong bands were sequenced immediately whereas weaker bands were re-amplified before sequencing by diluting 1 μl of the product 10–100-fold and using 1 μl of the dilution as the template in another round of PCR as described above. The DNA sequences were translated in silico using DNAsp v5.10.01 confirming the presence of the complete RRM amino acid sequence from –2 to +78 (predicted by PROSITE). Five *Ae. albopictus* samples from Northern Vietnam had identical RRM2 sequences [GenBank:KJ147319]. Among the *Ae. albopictus* samples from Central Laos (Khamcot), four samples had identical RRM2 sequences [GenBank:KJ147320], and one had an RRM1 sequence [GenBank:KJ147321]. Among the *Ae. albopictus* samples from Malaysia (Kuala Lumpur), three samples had identical RRM2 sequences [GenBank:KJ147317], one had an RRM1 sequence [GenBank:KJ147318.1] and one was heterozygous for RRM1 and RRM2. Two *Ae. polynesiensis* samples were obtained from Tahiti, but only one produced enough DNA for PCR and this carried the RRM1 sequence [GenBank:KJ147314]. Among the five *Ae. polynesiensis* samples from Fiji, three had identical RRM1 sequences [GenBank:KJ147316], one had an RRM2 sequence [GenBank:KJ147315] and one was heterozygous for RRM1 and RRM2.

Embryos from *Ae. aegypti* Rock mosquitoes were collected and a Femtotip II and FemtoJet microinjector system was used to inject the posterior of each embryo with the *tra-2* RNAi plasmid construct (1.6 μg/μl) to avoid the anticipated rapid *in vivo* degradation of dsRNA. Larvae were examined under a fluorescence stereomicroscope as above.

### Tests for reduced fertility and the analysis of spermatozoa in transgenic and natural meiotic drive (*MD*) mosquitoes

Two homozygous lines (2 and 10) were re-established from the heterozygous progeny of ♂M2 and ♂M10. Offspring from the intercrosses between heterozygotes were individually checked by PCR after mating and laying eggs. If both mother and father were homozygous, their eggs were used to establish the homozygous line. Males eclosed on the same day were kept in the same 2000-ml paper cup to accurately control for their age. Groups of five 3-day-old and 15-day-old males were collected from lines 2 and 10 (isofamily lines from line 6) and Rock. Each male was individually crossed with three virgin Rock females, i.e. 30 males and 90 females in total. From each set of three females, one was blood fed to lay eggs, one was dissected to examine the spermathecae immediately after coitus and the third one was dissected after 22 days.

Crosses were also set up between T37 males and drive-susceptible RED females. RED is a laboratory strain that carries multiple morphological mutant markers and was previously shown to be highly sensitive to meiotic drive. All three chromosomes of this strain contain genetic markers. T37 is a strain that carries a strong driver for meiotic drive. This strain originated from field samples collected from Trinidad [13]. F1 mosquitoes were intercrossed and sperm in the testes and spermathecae were tested. Images were captured using a DP50 digital camera connected to an Olympus BX51or ZEIZZ AXIOSKOP 2 microscope.

### Mosquito dissection and crossing experiments

Testes and spermathecae were dissected in *Drosophila* Ringer’s solution under a stereomicroscope. One testis or spermatheca was immersed in 12 μl Ringer’s solution on a slide and covered with a 24 x 50 mm coverslip. A pencil was used to press on the coverslip to release sperms from the organ and to distribute them onto the slide surface. Sperm activities were observed under an Olympus BH-2 microscope. The slide was cooled to –20 °C for 2 min to inactivate the sperm and was returned to the microscope stage for sperm counting under a grid eyepiece. In artificial mating experiments, females were mated in rapid succession [38] with transgenic and wild-type males to investigate the interaction between dysfunctional and healthy sperm within the same spermatheca. The first male was removed after 5–10 s of coitus to reserve space for a second male.

### Competitive mating experiments

Competitive mating between transgenic and Rock males was initiated by resting both types of males together in cages before releasing females.

### TRA/TRA-2 and *NvdsxRE* motif searches

We downloaded the expressed gene sequences of nine insects, namely *Ae. aegypti*, *An. gambiae*, *Cx. quinquefasciatus*, *D.melanogaster*, *Bombyxmori*, *Apis mellifera*, *Nasonia vitripennis*, *Triboliumcastaneum* and *Ixodes scapularis* from ftp://ftp.ncbi.nih.gov/repository/UniGene/. Data from seq.uniq.gz were used, although these include only one transcript from each gene and therefore may not contain all transcripts containing sex-specific splicing motifs. We also searched for these motifs in seq.all.gz which contains almost all the transcripts of each gene. However, all known transcripts remain to be updated in many important genes, e.g. *dsx*. We therefore decided to limit our results to the seq.uniq.gz search. Because each sex-specific splicing motif is usually distributed in a cluster of more than three units, we limited our search to genes containing a cluster of at least three patterns between 4 and 40 bp apart. The minimum distance default helps to avoid the detection of microsatellites, whereas the maximum distance default is used to ensure the sequences are clustered. We searched for *NvdsxRE* motifs [NGAAGAWN] in all nine species. We also searched for the TRA/TRA-2 motifs [39] found in *D. melanogaster* and *An. gambiae*, respectively [TC(T/A)(T/A)CAATCAACA] and [UCgcCgAUCAACc, cCAUCguUCAACc, UCAACA-UCAuCg, UCUcCAAUCAAuc, aCAUCAAUCAAuA, aCAUCAAUCAAuc].

### Statistical analysis

We used Python (http://www.compileonline.com/execute_python_online.php) for Χ^2^ analysis and created the corresponding charts using Microsoft Excel 2007.

## Results

### Comparison of *AAEL004293* RRM domains in the *Aedes* lineage

**Fig. 1:**
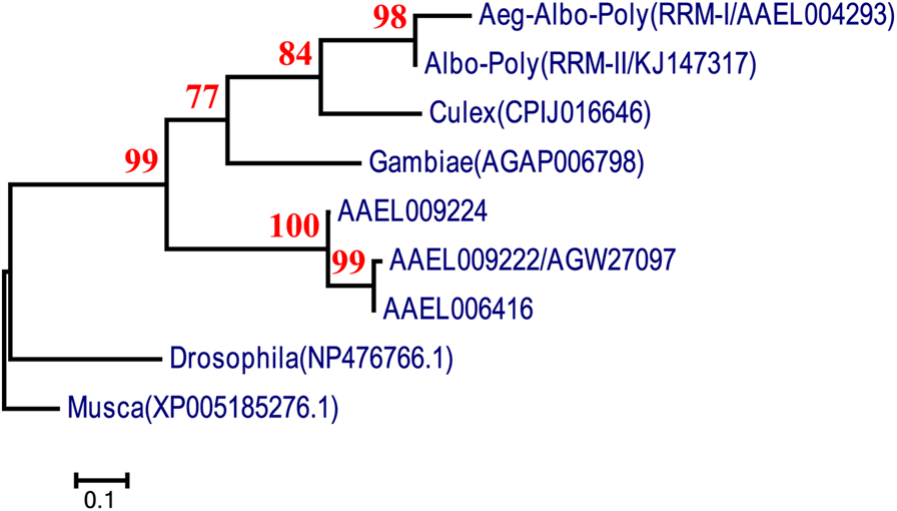
Phylogenetic tree derived from a maximum-likelihood analysis of TRA-2 RRM domain protein sequences. The phylogeny suggests that the duplication of *AAEL004293* probably occurred in a common ancestor of mosquitoes after the split of this ancestor from other diptera. The sequences of RRM domains were sourced from *Ae. aegypti (Aeg)*; *Ae. albopictus (Alb)*; *Ae. polynesiensis (Poly)*; *Cx. quinquesfaciatus (Culex)*; *An. gambiae (Gambiae)*; *Drosophila melanogaster* and *Musca domestica. AAAEL009222/AGW27097, AAAEL006416* and *AAAEL009224* are the three other *Ae. aegyptitra-2* homologs. The protein IDs for the RRM domains are shown on the branches of the phylogenetic tree. Bootstrap values are indicated in each node. The scale bar shows the branch length, representing the number of substitutions per site. Protein sequences in the phylogenetic tree can be found in the VectorBase and NCBI database with the following IDs: [VectorBase: *AAAEL009222/AGW27097, AAAEL006416, AAAEL009224 CPIJ016646, AGAP006798*]*,* [GenBan: *KJ147314, KJ147315, KJ147316, KJ147317, KJ147318, KJ147319, KJ147320, KJ147321, NP476766.1* and *XP005185276.1*]

RRM domains from *Ae. polynesiensis* and *Ae. albopictus* were amplified by PCR and characterized by sequencing, revealing the two haplotypes RRM1 and RRM2. These showed 100 and 88.46 % amino acid similarity, respectively, to the *AAEL004293* RRM domain of *Ae. aegypti* Rock. We also found RRM2 in two *Ae. polynesiensis* samples and RRM1 in three *Ae. albopictus* samples*.* A reconstructed maximum likelihood tree [34, 35] showed that *AAEL004293* RRM1 clustered with RRM2 and putative *tra-2* genes from *Anopheles gambiae* and *Culex quinquesfaciatus* rather than paralogs in *Ae. aegypti* (Fig. 1, Additional file 4: Figure S3 and Additional file 2: Table S2). We concluded that *AAEL004293* probably originated from a duplication of *tra-2* in a common ancestor of mosquitoes following the separation of mosquitoes from the remaining diptera.

### Investigation of the genetic mechanism of sex-determination

Transgenic line 6 was analyzed in genetic crossing experiments, which are summarized in Additional file 2: Table S3. In some crosses, the transgenic mosquitoes are described as *DsRed-2* males or females to distinguish them from non-transgenic mosquitoes in the same brood.

The transgene flanking sequence in line 6 was analyzed by inverse PCR (see methods) [37]. Template genomic DNA from line 6 (digested with Taq^α^I) was amplified using two pairs of nested inverse PCR primers, yielding products of 981 and 767 bp, respectively, suggesting only one insertion jumped into the line 6 genome. Additional PCR products should have been present after 35 cycles of the amplification if there was more than one insertion. A diagnostic PCR was used to establish homozygous colonies.

**Fig. 2:**
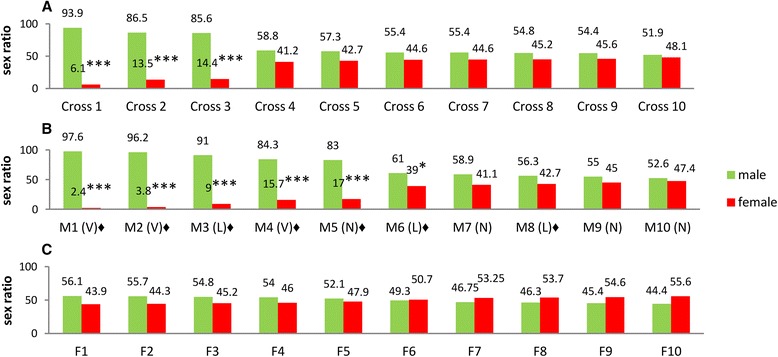
Determination of the sex ratio in *tra-2* RNAi transgenic mosquitoes. **a** Pupal sex ratio among the progeny of 10 single-pair crosses (Crosses 1**–**10) between homozygous males and females of line 6. **b** Pupal sex ratio among the progeny of 10 single-pair crosses (M1**–**10) between male (M) offspring from Cross-1 and Cross-2 and Rock females. Dissection of testes allowed males to be classified into those with very few sperms (V), lower sperm density (L) and normal sperm density (N) in comparison with Rock males. V = between 4 and 45 sperm; L = between 109 and 304 sperm; N = more than 400 sperm. **c** Pupal sex ratio among the progeny of 10 single-pair crosses (F1**–**10) between female (F) offspring of Cross-1 and Cross-2 and Rock males. The transgenic mosquitoes used for the crosses in **a**, **b** and **c** varied between 3 and 9 days old. Numbers at the top of the columns show the ratios of males and females. A significant deviation from the 1:1 sex ratio in a Chi-square test is indicated by *P < 0.05 or *** *P < 0.001*. The symbol ♦ indicates crosses in which dead sperms were found in the spermathecae of living Rock females 23 days after insemination by M1–10 males. All the crosses produced at least 80 eggs per cross with hatching rates >80 %, except for family F3 with a hatch rate of 71.9 %. The details of hatching rates and statistical tests are presented in the Additional file 5

Figure 2a shows male/female ratios in progenies of crosses between 10 pairs of homozygous parents ranging from 3 to 9 days in age (see methods). Interestingly, even in the absence of *t*TA, three of 10 crosses yielded predominantly males, with 94 % males in the most extreme case. The other seven crosses produced 1:1 sex ratios. Sex ratio distortion was not associated with a reduction in progeny size or hatching rate in any of the crosses. We therefore picked 10 random homozygous males (M1–10) and females (F1–10) from the progeny of the most male-biased crosses (Fig. 2a). These individuals were singly outcrossed with wild-type Rock females and males, respectively (Fig. 2b and c). Male ratios in the offspring produced by the M1–10 males showed a sex ratio bias similar to the previous generation, with five of the crosses producing 83–98 % males and the other five also yielding excess males but not to the same extreme (Fig. 2a and b) (see Additional file 5).

Sex ratio distortion was inherited only through the male parent. The hatching rates were >80 % in all the crosses, indicating that the male excess was not caused by significant female embryonic lethality. No sex ratio distortion was observed in the progeny of crosses between homozygous females and wild-type Rock males (Fig. 2c) suggesting the knockdown did not cause female-to-male sex conversion but that the distortion must be related to male gametogenesis.

The M1–10 parents were dissected to examine their spermatozoa. Three males produced only a few sperm, another three had a lower sperm count than normal and the remaining four had a sperm count similar to the Rock male controls. The Rock females crossed with the M1–10 males were blood fed for a second or third time to yield two further egg batches, and were dissected 22–23 days after mating to examine the sperm present in the spermathecae. In seven of the 10 females, almost all the sperm were dead and tended to aggregate when released into Ringer’s solution, and the few living sperm were motile (marked ♦ in Fig. 2b).

The between-family sex ratio variations shown in Fig. 2a and b, and the excess male progeny resulting from sperm depletion were strongly reminiscent of the outcome when *Ae. aegypti* males carrying the *D* gene linked to male-determining factor *M* were crossed with any female of a strain sensitive to the meiotic drive effect (*MD*), whose sons subsequently produced male-biased progeny [10, 11]. However, natural *MD* males show no reduction in their fertility unless mated sequentially to more than 15 females [12]. Moreover, mass mortality of sperms in the spermathecae has never been reported in cases of meiotic drive, and our recent *MD* tests for reduced fertility were negative (Additional file 2: Table S4) [13]. In the abovementioned test, offspring produced by crossing T37 males with RED females (see methods) were intercrossed in the F2 generation, and spermathecae dissected from females 22 days after intercrosses contained active sperms.

**Fig. 3:**
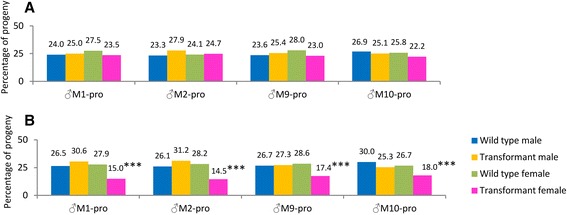
Segregation ratio of wild-type and heterozygous offspring in crosses between heterozygous male and wild-type mosquitoes at two different hatching times. Hatched eggs were produced from mass crosses between Rock females and heterozygous males (♂M1-pro, ♂M2-pro, ♂M9-pro and ♂M10-pro, the male progenies of M1, M2, M9 and M10). **a** One thousand eggs from each cross, produced after the first blood meal, showed no significant deviation from the anticipated 1:1:1:1 ratio in all the four mass crosses (*P > 0.05; df = 3; all X* ^*2*^ *≤ 5.73*). The hatching rates were in the range of 82.2–97.2 %. **b** Two hundred eggs produced as described in A but hatched 1 month later, showed a hatching rate of 74.5–97 % and pooled values of four mass crosses were significantly different from the anticipated 1:1:1:1 ratio due to the mortality of heterozygous females (^***^ *P < 0.001; all X* ^*2*^ *≥ 24.20; df = 3*). After removing the heterozygous females from the counts, pooled values of heterozygous males, wild-type males and females in B did not deviate significantly from the anticipated 1:1:1 ratio (*P > 0.05; X* ^*2*^ *= 0.00; df = 2*). The numbers at the head of each column show the sex ratios of transgenic and wild-type mosquitoes from the same broods. The transgenic mosquitoes used for these crosses varied between 3 and 9 days old. The details of hatching rates and statistical tests are presented in Additional file 5

In the *MD* systems, successive crosses to sensitive Rock females should produce male-biased progeny [10, 11, 40]. To test whether the heterozygous sons from homozygous fathers had been subjected to drive via the *MD* system, we crossed 25 heterozygous males from the biased progenies of M1 and M2, and the unbiased progenies of M9 and M10, with 25 virgin wild-type Rock females (Fig. 2b). All the mass crosses showed a 1:1 sex ratio and a 1:1:1:1 segregation ratio for wild-type males, *DsRed-2* males, wild-type females and *DsRed-2* females (*P > 0.05; all X*^*2*^ *≤ 5.73; df = 3*) as shown in Fig. 3a (see Additional file 5).

There was no deviation from the anticipated 1:1 ratio when testing the overall or male (*DsRed-2*): wild-type ratio or the male (*DsRed-2*):female(*DsRed-2*) ratio, confirming again that there was a single transgene insertion, that none of the 25 males were chromosomal karyotype mm, and that the jumping site in line 6 was an autosome. There was also no female-to-male sex conversion as found in other dipterous insects. Because the knockdown of *tra-2* does not cause sex conversion even though TRA/TRA-2 regulatory elements are present in female-specific *dsx* pre-mRNA sequences [29], *tra-2* clearly does not play the same role during sex determination in *Ae. aegypti* as in certain other dipterous insects [18–20].

Interestingly, 200 eggs from the first blood meal batches were hatched a month later and showed a reduction in the proportion of transgenic females (Fig. 3b), resulting in a significant deviation from the anticipated 1:1:1:1 ratio of wild-type males, heterozygous males, wild-type females and heterozygous females (*P < 0.001; X*^*2*^ *= 24.20; df = 3*). Even in the crosses using the ♂M9 and ♂M10 progeny, in which the sex ratio was unbiased in the first generation (Fig. 2b), the pooled value was significantly distorted in the later-hatching egg batches of the subsequent generation (*P < 0.05; X*^*2*^ *= 8.99; df = 3*) as shown in Fig. 3b.

The differences in sex ratio in the later-hatching egg batches provide convincing evidence against the presence of natural *MD* drive. The loss of females was confined entirely to the transgenic population (*P < 0.001*), and if these females are excluded there was a 1:1:1 ratio of wild-type males, *DsRed-2* males and wild-type females (*P > 0.05; X*^*2*^ *= 0.00; df = 2*) (see Additional file 5). If natural *MD* drive contaminated or segregated to the insertion in line 6, it would skew the progeny sex ratio of both the transgenic and wild-type females [10, 11]. The sequences flanking the insertion in line 6 matched an intergenic region near the *Odorant Receptor-84* gene [AaegL1:supercont1.110], which is unlikely to be an *MD* enhancer. This supercontig has yet to be mapped onto a chromosome, but the genetic crosses described above confirmed that supercont 1.110 is not a sex-linked region in *Ae. aegypti* chromosome I.

Evidently this sex ratio distortion is caused by the transgene, because even in the absence of *t*TA, the leaky *tet*O sequence [41, 42] allowed sufficient residual expression of the *tra-2* RNAi construct to inhibit m-chromosome-bearing sperm and also mm zygotes, thus increasing the proportion of male offspring.

### Sperm longevity, male age relative to sex ratio distortion and male competence

**Fig. 4:**
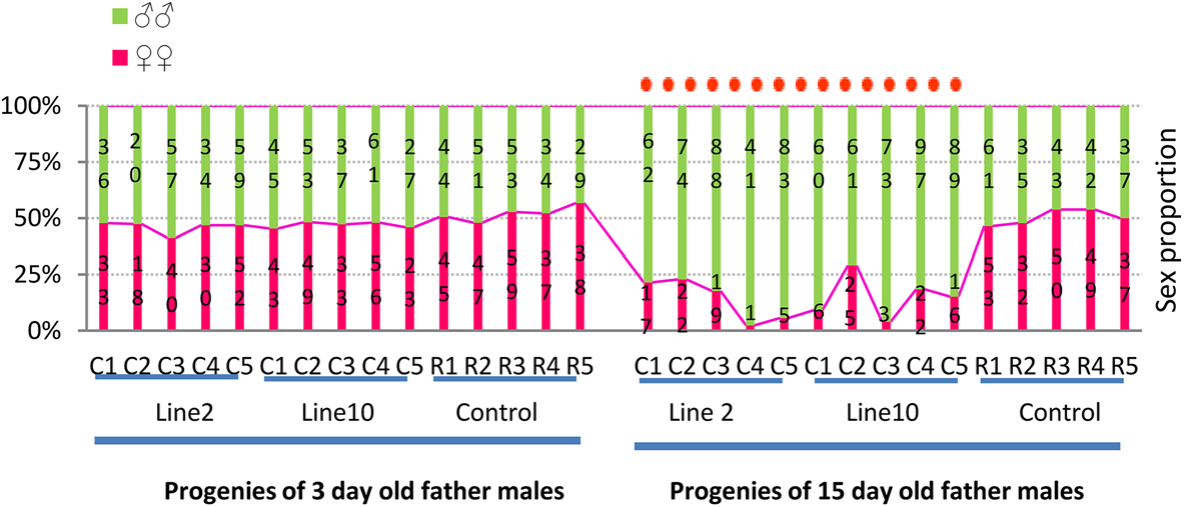
Transgenic male ages in relation to sex ratio in the next generation. Transgenic homozygous males (C1, C2, C3, C4 and C5) from lines 2 and 10 and control Rock males (R1, R2, R3, R4 and R5) were divided into 3-day-old and 15-day-old age groups. The males in each group were crossed with Rock females. C1-5 and R1-5 are single-pair crosses. The stacked numbers in each column are values observed in each cross. The 3-day-old parental males produced no significant deviation from the anticipated 1:1 sex ratio. The red dashed line indicates 10 crosses showing significant deviation from the anticipated 1:1 sex ratio (*P < 0.001; df = 1; all X* ^*2*^ *≥ 15.07*) when parental males were 15 days old. The details of hatching rates and statistical tests are presented in Additional file 5

The crossing and dissection experiments indicated an association between sperm longevity, sex-specific lethality and transgene expression. To test the consistency of those factors, two homozygous lines (2 and 10) were re-established from the heterozygous progeny of ♂M2 and ♂M10 (Fig. 4). ♂M2 produced highly-biased male offspring whereas the offspring of ♂M10 were unbiased. Groups of five 3-day-old and 15-day-old males were collected from lines 2, 10 and Rock, and each male was individually crossed with three virgin Rock females. One of the three was blood fed to lay eggs, the second was dissected to examine the spermathecae immediately after coitus and the third was dissected after 22 days.

**Fig. 5:**
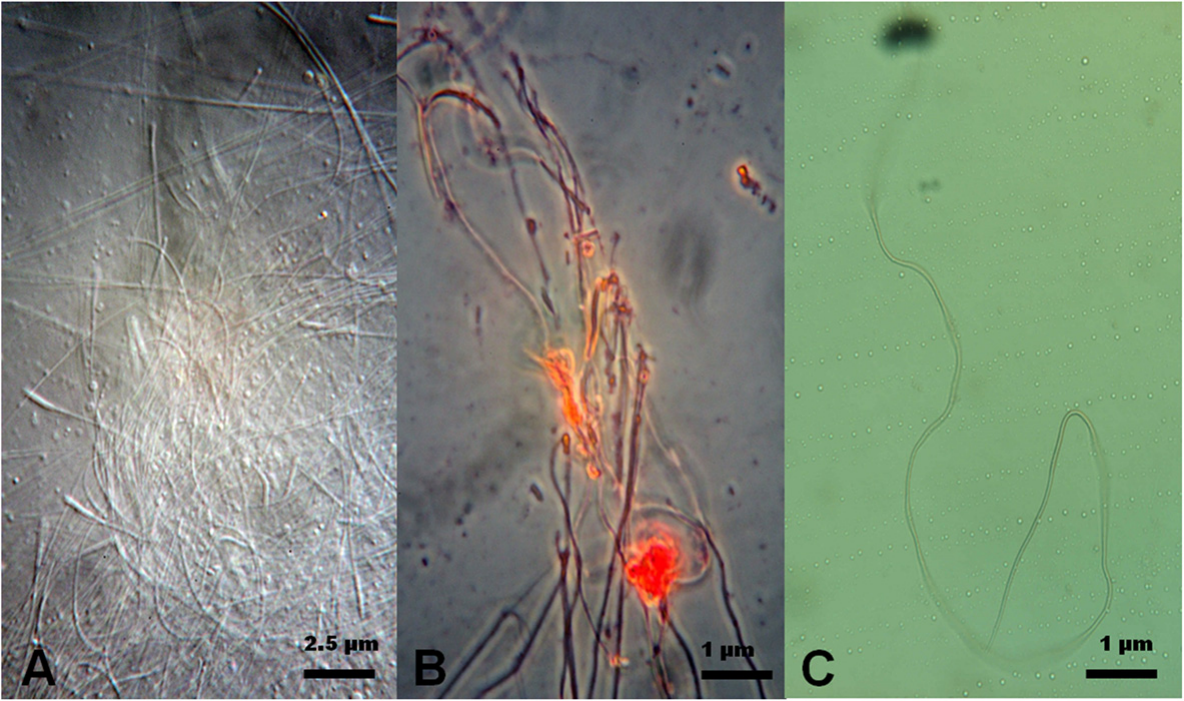
Dead sperm in the spermathecae of female mosquitoes. **a** Dead sperm without staining. Scale bar = 2.5 μm. **b** Dead sperm stained with Orcein. Scale bar = 1 μm. **c** Stained sperm from the age-matched control wild-type males. Scale bar = 1 μm. Images captured with a DP50 digital camera fitted to an Olympus BX51 microscope

In all crosses, the 3-day-old fathers produced normal sperm and 1:1 sex ratios in the offspring (Fig. 4; Additional file 2: Table S4). Active and normal sperm were also found in the spermathecae of the mated females. However, the 15-day-old fathers produced a lower density of sperm, and the sex ratios were significantly skewed from 1:1 (*P < 0.001; all X*^*2*^ *≥ 15.1*; *df =1*) (see Additional file 5). The sperm in the spermathecae of females crossed with 15-day-old males were present at a lower density than normal, but dead sperm were not found because these cannot pass into the spermathecae. The presence of sperms that died after entering the spermathecae indicated that the *tra-2* knockdown effect may be weak, even in homozygous males. No difference in sperm vitality was observed among the Rock males regardless of age. The spermathecae of females kept for 22 days after mating contained predominantly dead sperm after crossing with lines 2 and 10, whereas living sperm were found in those crossed with Rock males (Fig. 5; Additional file 2: Table S4).

Our results show that *tra-2* RNAi causes the haplotype-specific differential depletion of sperm, and that lethality increases with age. The high mortality observed for almost all the sperms in the spermathecae may indicate that the decomposition of dysfunctional m-chromosome-bearing sperm has a toxic effect on those carrying the M-chromosome. Interestingly, the *MD* effect in *Ae. aegypti* involved an increase in sperm senescence as well as moderate post-zygotic mortality. In the extreme case, *MD* affects most sperm, but normal fertility requires no more than 20 % of typical sperm production in *Ae. aegypti* [10, 43]. In *D.simulans*, X^K^s gene not only killed wild-type sperm but many X^K^s sperm were autolethal [44, 45]. The mass sperm mortality observed in the *Ae. aegypti tra-2* knockdown line may therefore represent an evolutionary strategy similar to natural *MD*, especially given the genetic similarity between the *Ae. aegypti* m-chromosome and M-chromosome. We also tested the mating capacity of transgenic males in competition experiments and found they competed as well as wild-type males under cage conditions (Additional file 2: Table S5). We also searched nine insect transcriptomes for *NvdsxRE* motifs [29] and found that the sequences were prevalent in these species suggesting widespread evolutionary significance (Additional file 2: Table S6).

### Microinjection of the *tra-2* RNAi construct: transient effect on sex ratios

The microinjection of *tra-2* dsRNA surprisingly produced no effect in *Ae. aegypti* [27]. To prevent the rapid *in vivo* degradation of dsRNA, we injected *Ae. aegypti* Rock embryos with the *tra-2* RNAi plasmid construct at a high concentration of 1.6 μg/μl. The survivors showed a significant male bias ratio but these males produced a normal sex ratio in subsequent generation, indicating that transient expression of the construct killed female zygotes independent of genetic background or transgene integration effects (Additional file 2: Table S7).

### Segregation distortion and *Ae. aegypti* sex determination

**Fig. 6:**
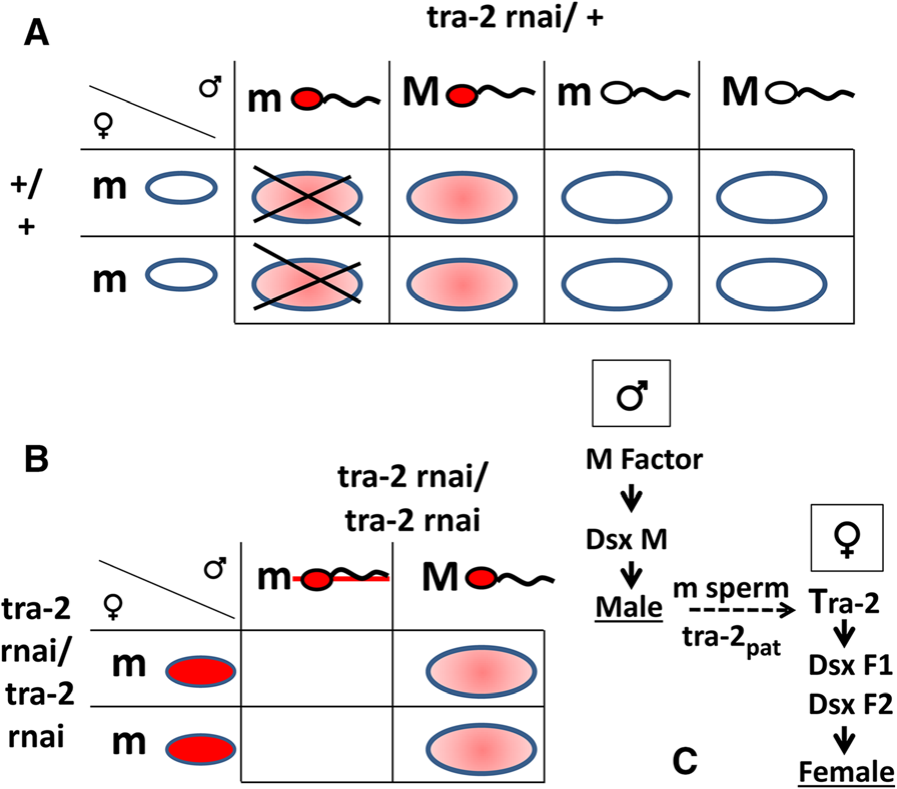
Female-specific lethality and sex determination. **a** Punnett square showing *tra-2* RNAi segregation in a cross between a heterozygous male and a wild-type female. The knockdown effect driven by the minimal *CMV* promoter was not enough to cause an obvious effect on sperm activity but specific lethality in mm zygotes was still evident. **b** Punnett square showing *tra-2* RNAi segregation in a homozygous line, resulting in dysfunctional m-chromosome-bearing sperm during spermatogenesis and no surviving female progeny after fertilization. **c** Hypothetical model of sex determination in *Aedesa egypti* in which paternal *tra-2* mRNA (tra-2_pat_) is necessary for the activity of m-chromosome-bearing sperms

Our results show that *Ae.aegypti tra-2* is responsible for a *SD* mechanism (Fig. 6a and b). Sperms carrying the m-chromosome require paternal *tra-2* to function correctly, suggesting that *tra-2* is potentially a master-control locus for sex-determination which induces the specific inactivation of sperm carrying the m-chromosome. We propose a model in which the TRA/TRA-2 complex controls the splicing of *dsx* mRNA, leading to the production of two female-specific isoforms (DSX^F1^ and DSX^F2^) and the male-specific isoform DSX^M^. During zygotic development, *dsx*F1 is not synthesized in the embryo or in the first and second larval stages whereas *tra-2* knockdown would prevent the synthesis of *dsx*F2 [29]. The mm genotype is therefore zygotic lethal as found in our experiments (Fig. 6c). The lack of sex conversion indicates that the reversible sex determination of this species is not a maternal decision.

## Discussion

We investigated a transgenic *Ae. aegypti* line carrying an RNAi construct that specifically targets the *tra-2* gene. The analysis of this line in large-scale crosses indicated that the knockdown of *tra-2* is probably responsible for the dysfunction of m-chromosome-bearing sperm and female-specific zygotic lethality in the subsequent generation, producing all-male progenies. Although we cannot distinguish dead (m) and living (M) sperm, the mass mortality of sperm and accompanying male bias suggests that *tra-2* knockdown causes *SD*. The knockdown of *tra-2* in transgenic males did result in the depletion of *tra-2* (*AAEL004293*) mRNA, but quantitative RT-PCR analysis of mRNA from the testes of individual transgenic fathers would provide additional confirmatory data [30]. The homozygous murine Rb (6.15) *Spam1* mutation caused a reduction in mRNA levels (to 45 % of the wild-type level) and heterozygous mice carrying this mutation produced two populations of sperm, one with the mutation being unable to bind to the zona of the egg [46]. In line 6, the *tra-2* expression level in male homozygotes was reduced by an average of 48.7 % relative to wild-type insects (*Standard Deviation = 4.98*) [30]. These knockdown levels were not enough to cause sperm dysfunction in heterozygous males, hence the resulting 1:1:1:1 ratio (Additional file 3: Figure S2 and Fig. 3a).

The genetic construct in line 6 behaves oddly because the knockdown occurred even in the absence of *t*TA and only some of the crosses showed evidence of *SD* (Fig. 2a and b). The incomplete penetrance of the *SD* effect could be caused by the genetic background. However, in ♂M9 and ♂M10 crosses, the sex ratio was unbiased but the male offspring produced significantly distorted sex ratios if their egg batches were hatched a month later (Fig. 3b). Homozygous 3-day-old males produced 1:1 sex ratios in their offspring but 15-day-old homozygous males consistently produced distorted ratios. The Rock line, which was used to create line 6, consistently showed a 1:1 sex ratio (Fig. 4). Altogether, these results clearly exclude genetic background effects on the degree of *SD* and indicate that there was a linear relationship between the residual expression of the *tra-2* RNAi construct and the age of males.

No sex ratio distortion was found in the progeny of crosses between homozygous females and wild-type Rock males (Fig. 2c) but crosses between homozygous *tra-2* RNAi females (Additional file 2: Table S1) [30] and homozygous *LA513* males [33] carrying the *t*TA transactivator gene yielded a significantly biased sex ratio (up to 70 % males) in the absence of tetracycline, indicating that the abnormal sex ratio can be attributed to the fully penetrative activation of the *t*TA promoter. These results are consistent with our finding that heterozygous female mortality occurred in the later-hatching egg batches and in the transient expression experiments (Fig. 3b and Additional file 2: Table S7). Altogether, these results indicate that *tra-2* RNAi female zygotes can be killed at early stages of development if the knockdown effect is potent, and that the *tet* transactivator system is suitable for the conditional induction of *tra-2* RNAi [31, 47].

Theoretically, an autosomal driver acting on sex-chromosomes cannot undergo positive selection because the autosomes would be selected to re-establish an equal sex ratio [48]. Endogenous retrotransposon-dependent RNAi has been shown to cause transmission ratio distortion in *D. simulans* [14, 15] and the multiple *tra-2* loci in mosquitoes may have been exploited in a similar manner [49]. The striking similarity between our RNAi phenotype and the natural *MD* system in terms of sperm depletion/dysfunction, between-family sex ratio bias, egg hatching rates and zygotic lethality [10, 11, 43], may indicate the presence of such a genetic element within the *D* gene and the multiple *tra-2* loci may have been selected to counteract the natural *MD* system [49]. A translocation from chromosome III to the M-chromosome in *Ae. aegypti* confirms that genetic dominance is not responsible for the *MD* phenomenon [10]. Our findings strongly support the similarity between *tra-2* knockdown and natural *MD* in Culicine mosquitoes [10–13, 40].

The *tra-2* knockdown provides opportunities for the future development of novel vector control strategies. Strongly biased male progeny may encourage the conditional elimination of females, allowing the efficient production of genetic sexing strains that could be used for mosquito control based on SIT [1]. If the *tra-2* RNAi construct were linked to the M-chromosome and expressed under a suitable spermatogenesis-specific promoter such as *β2* [50, 51], the dsRNA could be used to target all m-chromosome-bearing sperm [52, 53] and rapidly masculinize a target population [54, 55]. This is not a purely speculative proposal because 15-day-old homozygous males produced all biased offspring but heterozygous males produced offspring with normal Mendelian segregation, suggesting that a powerful spermatogenesis-specific promoter may cause *SD* in the offspring with 100 % penetrance whether the father is homozygous or heterozygous. A suppressor RNAi construct that can inhibit *t*TA gene expression in the M linked *tra-2* RNAi construct could also be used as a vehicle to carry new genes into a population [56].

A recent article [57] has reported that feeding mosquito larvae with dsRNAs targeting testis-specific genes produces adult males with greatly reduced fertility. RNAi-mediated knockdown of the female-specific isoform of *dsx* also induces female lethality and a highly-male-biased population of mosquitoes. This *dsx* knockdown-dependent female lethality is consistent with our observed female-specific zygotic lethality, given the interaction between *dsx* and *tra-2*, and this finding could revolutionize the SIT method. For example, *Glossina* spp. (tsetse flies) are viviparous, preventing the application of conventional transgenic methods, but dsRNAs feeding may allow the application of SIT [58].

If mosquito larvae are fed *tra-2* dsRNA, a male-biased population would be anticipated with few females surviving until adulthood. The advantage of a conditionally-transgenic *tra-2* RNAi system is that specific promoters could be used to restrict the fatal effect of *tra-2* RNAi solely to the m-sperm, leaving the females intact. Eggs produced by such transgenic lines should give rise to males only, which could be stored in a conventional manner and delivered when necessary within a few months, thus avoiding the need to rear millions of male insects per week. The first major success of a mosquito SIT program was achieved against *Cx. pipiens fatigans* in Myanmar [59], in which SIT males sterilized wild female counterparts by carrying different strains of Wolbachia to those carrying by the natural mosquito populations. This can be applied to minimize concerns coupled with the release of transgenic organisms, the sterility of the released males would ensure biological containment and prevent the spread of transgenes. If a transgenic *Ae. aegypti* line carrying *wMel* Wolbachia was allowed to mate wild females, no offspring would be produced due to their complete cytoplasmic incompatibility [60, 61].

## Conclusion

We have investigated the function of the *tra-2* gene in an *Ae.aegypti tra-2* RNAi transgenic line. We found that the knockdown of *tra-2* gene expression causes a bias in the sex ratio of the next generation due to segregation distortion acting at the level of gametic function and female-specific zygotic lethality. All 15-day-old homozygous fathers produced significantly male biased progeny, which indicates similar penetration of the genetic construct in age-matched males and that the *tra-2* RNAi construct could be used as a sex distorter system. A fully conditional *tet* regulatory system would be needed to establish *Ae. aegypti* genetic sexing strains that could be integrated into control techniques based on SIT or RIDL.

## Availability of supporting data

The data sets supporting the results in this article are included within the article and its additional files.

## Additional files

Additional file 1: Figure S1. Construct schematic. Schematic representation of the Zoo-2 vector [30], linearized at the 3′ end of the *PiggyBac* transposon. The inserted sequences enhance the efficiency of intron splicing. The two functional segments include the marker (*Act5C* promoter, *DsRed-2*, *Drosomycin* 3′-UTR) and a *tra-2* RNAi cassette (*tet*O7-CMV minimal promoter, RRM inverted repeats joined by the *tra-2* intron and rabbit β-globulin 3′-UTR). (TIF 1813 kb) Additional file 2:
**Supporting Information in Tables.** (DOCX 38 kb) Additional file 3: Figure S2. The expression of *tra-2* in wild-type males and *tra-2* RNAi transgenic males. Quantitative real-time PCR was also used to detect the level of *tra-2* mRNA remaining in homozygous male pupae of line 6 (red) and wild-type Rock male pupae (blue). The *tra-2* expression level was reduced by an average of 48.7 % in line 6 relative to wild-type insects in three replicates (*Standard Deviation = 4.98*) [30]. (TIF 8549 kb) Additional file 4: Figure S3. Schematic representation of the homologous TRA-2 proteins. (A) Protein encoded by the *AAEL009224* gene located on supercontig 1.381. (B) Protein encoded by the *AAEL009222* gene located on supercontig 1.381. (C) Protein encoded by the *AAEL006416* gene located on supercontig 1.204. (D) Protein encoded by the *AAEL004293* gene located on supercontig 1.113. These proteins were annotated by merging Ensembl and TIGR prediction sets in Vector Base: Aedes aegypti. (E) RNAi target site in the RRM region of the *AAEL004293* gene. (TIFF 5036 kb) Additional file 5: The accession numbers of the RRM sequences: [GenBank: KJ147314, KJ147315, KJ147316, KJ147317, KJ147318, KJ147319, KJ147320, KJ147321]. (XLSX 26 kb)
