# Supplementary figures and images for: Mechanisms of sex determination and transmission ratio distortion in Aedes aegypti
Source: Parasit Vectors. 2016 Jan 28;9:49. doi: 10.1186/s13071-016-1331-x (PMC4730765; doi:10.1186/s13071-016-1331-x)

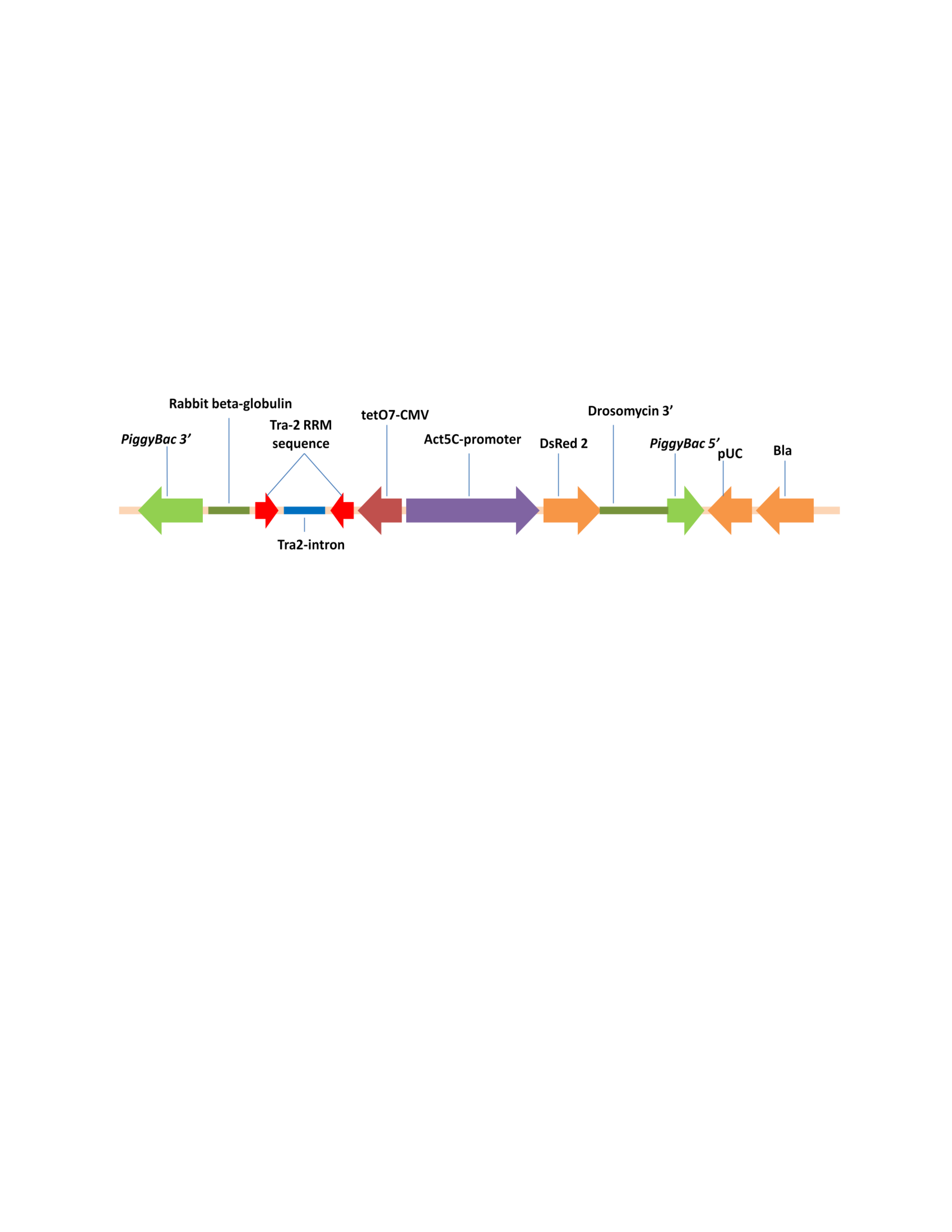

Supplement: Additional file 1: Figure S1. — Construct schematic. Schematic representation of the Zoo-2 vector [30], linearized at the 3′ end of the PiggyBac transposon. The inserted sequences enhance the efficiency of intron splicing. The two functional segments include the marker (Act5C promoter, DsRed-2, Drosomycin 3′-UTR) and a tra-2 RNAi cassette (tetO7-CMV minimal promoter, RRM inverted repeats joined by the tra-2 intron and rabbit β-globulin 3′-UTR). (TIF 1813 kb) [file 13071_2016_1331_MOESM1_ESM.tif]
