# Supplementary material for: Mechanisms of sex determination and transmission ratio distortion in Aedes aegypti
Source: Parasit Vectors. 2016 Jan 28;9:49. doi: 10.1186/s13071-016-1331-x (PMC4730765; doi:10.1186/s13071-016-1331-x)
Supplement: Additional file 2: — Supporting Information in Tables. (DOCX 38 kb) [file 13071_2016_1331_MOESM2_ESM.docx]

**Mechanisms of sex determination and transmission ratio distortion in *Aedes aegypti***

***Kim Phuc Hoang et al***

# Supporting Information

**Suppl. Table 1. Crosses between the *tet*-responsive line 6 and the Tet-off *t*TA line *LA513*.**

| Cross | Total embryos | Percentage hatch  (larvae/ embryos) | Percentage Pupation | Percentage male | *Χ^2^_test*_* |
| --- | --- | --- | --- | --- | --- |
| +Tet | 246 | 97% (238/246) | 99% (235/238) | 52% (122/235) | 0.072 (*p = 0.951*) |
| –Tet | 163 | 96% (156/163) | 53% (83/156) | 70% (58/83) | 7.904 (*p = 0.005*) |

Homozygous transgenic females (*tra-2* RNAi) were crossed with *LA513* homozygous males carrying the *t*TA locus [30]. Eggs were hatched in two conditions in the presence (+Tet) and absence (–Tet) of tetracycline. **Χ^2^*^­^ test for deviation from sexual parity in pupae (*P<0.01* for the –Tet cross, *X^2^ = 7.904, df = 1*).

**Suppl. Table 2.** **Net evolutionary divergence between groups of RRM sequences.**

|  | [1 | 2 | 3 ] |
| --- | --- | --- | --- |
| [1] |  | [ **0.073**] | [ **0.077** ] |
| [2] | **0.370** |  | [ **0.107** ] |
| [3] | **0.359** | **0.540** |  |

#**Group-1**

[1] #Aeg-Albo-Poly(RRM-I : AAEL004293/KJ147318/KJ147321/KJ147316/KJ147314)

[2] #Abo-Poly(RRM-II: KJ147317/KJ147320/KJ147315)

[3] #Culex quinquefasciatus (CPIJ016646)

[4] #Anopheles gambiae (AGAP006798)

#**Group-2**

[5] #AGW27097/AAEL009222

[6] #AAEL006416

[7] #AAEL009224

#**Group-3**

[8] #Drosophila melanogaster (NP476766.1)

[9] #Musca domestica (XP005185276.1)

Estimates of net evolutionary divergence between groups of sequences (RRM regions from putative *tra-2* homologs of mosquitoes and two other dipteran insects) using the Poisson correction model in MEGA5 [34, 35]. The number of amino acid substitutions per site is averaged across all sequence pairs among groups. There were 1000 bootstrap replications. Standard error estimates are shown above the diagonal. The rate variation among sites was modeled with a gamma distribution (shape parameter = 1). The analysis involved nine amino acid sequences. All positions containing gaps and missing data were eliminated. A total of 78 positions were included in the final data set.

**Suppl. Table 3. Order of the crosses for genetic analysis.**

| **Line 6 was genetically analyzed by the crosses below**  ***A. 10 ♀♀ i/i* x *10 ♂♂ i/i [Cross 1 to Cross 10]***  ***B. 10 ♂♂ i/i (from Cross 1 & 2)*  x 10 *♀♀ Rock +/+ [M1 to M10]***  ***C. 10 ♀♀ i/i (from Cross 1 & 2)*  x 10 *♂♂ Rock +/+ [F1 to F10]*** | **Fig. 2 A**  **Fig. 2 B**  **Fig. 2 C** |
| --- | --- |
| ***D. 25 ♂♂ M1-pro i/+ (progeny of M1)* x 25 *♀♀ Rock i/i***  ***E. 25 ♂♂ M2-pro i/+ (progeny of M2)* x 25 *♀♀ Rock i/i***  ***F. 25 ♂♂ M9-pro i/+ (progeny of M9)* x 25 *♀♀ Rock i/i***  ***G. 25 ♂♂ M10-pro i/+ (progeny of M10)* x 25 *♀♀ Rock i/i*** | **Fig. 3A. First blood meal eggs hatch 3 days old.**  **Fig. 3B. First blood meal eggs hatch 30 days old.** |
| **M2-pro *i/+* and M10-pro *i/+* were re-established into two**  **homozygous lines (2 and 10)**  ***H. ♂♂ line 2 i/i x Rock +/+ ♀♀***  ***I. ♂♂ line 10 i/i x Rock +/+ ♀♀***  ***J. ♂♂ Rock +/+ x Rock +/+ ♀♀*** | **Fig. 4, Suppl. Table 4** |
| **Meiotic drive crosses as controls to check sperm longevity**  ***K. ♂♂*T37 *MD/mD* x RED *md/md ♀♀* F1**  ***L ♂♂MD/md x mD/md ♀♀* F2** | **Suppl. Table 4** |

The inserted *tra-2* RNAi construct is shown as *i*, natural M-chromosome meiotic drive is shown as *MD* and the sensitive m‑chromosome is shown as *md* [10, 11]. (**A-C**) Crosses for clarification of the sex ratio in the *tra-2* RNAi transgenic mosquitoes. (**D-G**) Crosses to reveal segregation ratio of wild-type and heterozygous offspring in crosses between heterozygous male and wild-type mosquitoes at two different hatching times. (**H-J**) Crosses to detect the relationship between transgenic paternal ages and the sex ratio of the next generation. (**K-L**) Crosses to observe sperm activities from meiotic drive males (progeny of ♂T37 x *♀*RED) after insemination of spermathecae at different ages. All the details of egg numbers, hatching rates, survival rates and statistical tests are presented in Additional File 2 (Original cross data.xlsx).

**Suppl. Table 4.** **Sperm activities in the testes and spermathecae at different ages**.

| Line | | Sperm in  3-day-old male testes | | Sperm in  15-day-old male testes | | Sperm in  spermathecae  after coitus with | | | | Sperm in  spermathecae  22 days after coitus with | | | |
| --- | --- | --- | --- | --- | --- | --- | --- | --- | --- | --- | --- | --- | --- |
|  | |  | |  | | ♂3 days old | | ♂15 days old | | ♂3 days old | | ♂15 days old | |
| **2** | | 5 | N, A | 4  1 | L, A  V, A | 5 | N, A | 4  1 | L, A  NS | 1  3  1 | Mix  D  n/a | 4  1 | D  Mix |
| **10** | 5 | N, A | 5 | L, A | 5 | N, A | 5 | L, A | 2  3 | Mix  D | 4  1 | D  n/a |  |
| **Rock** | | 5 | N, A | 5 | N, A | 5 | N, A | 5 | N, A | 5 | N, A | 4  1 | N, A  n/a |
| **F1*** | | 10 | N, A | 10 | N, A | 10 | N, A | 10 | N, A | 10 | N, A | 15 | N, A |

N = normal sperm density; L = lower sperm density; V = very few sperm; NS = no sperm; A = active sperm; D = dead sperm; n/a = female dead; Mix = very few vital sperm + dead sperm; * = F1 progeny of ♂♂T37 x Red***♀♀.*** V counts indicate observations between 4 and 45 sperm in testes or spermathecae; L counts indicate observations between 109 and 304 sperm in testes or spermathecae; N counts indicate observations of more than 400 sperm in testes or spermathecae; Dead sperm in spermathecae clump together preventing accurate counting. Our counting method for total sperm from a testis or spermatheca allows the detection of extremely low numbers of sperm in each testis or in the spermathecae of females crossed to transgenic males. When the numbers of sperm were more than 400 as usually found in the control testes or spermathecae, overcrowding prevented accurate counting. We assumed that more than 400 sperm in the control testes or spermathecae is normal density.

**Suppl. Table 5.** **Male competitive mating data.**

| Replicates | Mass cross  (in 0.4 x 0.4m cages) | Wild-type offspring | *DsRed*-2 offspring | Χ^2­^ test for   deviation |  |
| --- | --- | --- | --- | --- | --- |
| 3 | 25 ♂*tra-2* (homo)  25 ♂ wild type  25 ♀ wild type | 88  91  86 | 80  82  85 | *P > 0.05*  *P > 0.05*  *P > 0.05* | 1:1 |
| 3 | 25 ♂ *tra-2* (hetero)  25 ♂ wild type  25 ♀ wild type | 121  131  123 | 43  41  36 | *P > 0.05*  *P > 0.05*  *P > 0.05* | 3:1 |

Three-day-old males (Rock, *tra-2* RNAi homozygotes and *tra-2* RNAi heterozygotes) were rested in cages before Rock females were introduced. We collected 200 eggs from each cage for hatching.

**Suppl. Table 6. Relative frequency of TRA/TRA-2 and *NvdsxRE* cluster motifs in gene transcripts from nine insect taxa.**

| Species | TRA/TRA-2 |  | *NvdsxRE* |  | Motif in *dsx* |
| --- | --- | --- | --- | --- | --- |
| *Ae. aegypti* | 16663=0 (0.99)  17≤ 2 (0.00) |  | 5525= 0 (0.33) 10873 ≤ 2 (0.65)  282 ≥ 3 (0.02) |  | DQ440532*  TRA/TRA-2  *NvdsxRE* |
| *An. gambiae* | 13062=0 (0.99)  4≤ 2 (0.00) |  | 6305 = 0 (0.48)  6714 ≤2 (0.51)  47≥3 (0.01) |  | DQ137802  TRA/TRA-2** |
| *Cx. quinquefasciatus* | 5020=0(0.99)  1≤ 2(0.00) |  | 1484 =0 (0.30)  3453 ≤2 (0.68)  84≥3 (0.02) |  | CPIJ004058  Neither |
| *D. melanogaster* | 17138=0(0.99)  18≤ 2(0.00) |  | 5930 =0 (0.34)  11090 ≤2 (0.65)  136 ≥3 (0.01) |  | NM_079548  TRA/TRA-2 |
| *B. mori* | 13943=0(0.99)  9≤ 2(0.00) |  | 5058 = 0 (0.36)  8706≤ 2 (0.63)  188 ≥ 3 (0.01) |  | AB048543  Unique |
| *A. mellifera* | 24376=0(0.99)  16≤ 2(0.00) |  | 7718 = 0 (0.33)  15147 ≤ 2 (0.64)  627 ≥3 (0.03) |  | NM_001134935  *NvdsxRE* |
| *N. vitripennis* | 15428=0  17≤ 2(0.00) |  | 7271=0 (0.47)  7926≤2 (0.51)  248 ≥ 3 (0.02) |  | NM_001162517  *NvdsxRE* |
| *T. castaneum* | 6847=0(0.99)  5≤ 2(0.00) |  | 2609 = 0 (0.38)  4063 ≤ 2 (0.59)  180 ≥ 3 (0.03) |  | JQ857099  *NvdsxRE* |
| *Ix. scapularis* | 19398=0(0.99)  7≤ 2(0.00) |  | 9511 = 0 (0.49)  9839 ≤ 2 (0.51)  55≥3 (0.00) |  | XM_002403405  Neither |

Number (0, 2, 3) refers to the number of motif units found in each mRNA sequence; * = GenBank reference sequence; ** = motif sequence degradation. (<ftp://ftp.ncbi.nih.gov/repository/UniGene/>).

**Suppl. Table 7**. **Survival and sex ratio of *Ae. aegypti* Rock embryos after microinjection with *tra-2* RNAi plasmid construct.**

| Construct | Injected embryos | Survival rate (%) | Female | Male | Χ^2^_test_ |
| --- | --- | --- | --- | --- | --- |
| *tra-2* RNAi | 700  400 | 132 (18.9)  84 (21.0) | 53  33 | 79*  51 | *P<0.05 (5.12)*  *P<0.05 (3.86)* |

Pooled data were tested for deviation from sexual parity, *P < 0.005, X^2^ = 8.092, df = 1*; * = 15 males were singly crossed with wild-type females producing a 1:1 sex ratio (*P > 0.05*, *X^2^* ranges between *0.04* and *2.33*, *df = 1*).

**Suppl. Table 8**. **Primer sequences used to assemble the *tra-2* RNAi construct.**

| 1-(SacII-Ex1F)  2-(XhoI**-**Ex1R)  3-(BamHI-Ex1F)  4-(EcoRI-Ex1R)  5-(XhoI-Int-F)  6-(EcoRI-Int-R1) | 5'-cgatctcccgcggATGCCAAGACGAAGGTTTCCCGAG-3'  5'-Cggcaatgacctcgagcggtcaccgaataatccactcaa-3'  5'-GGCgtcaatgGATCcATGCCAAGACGAAGGTTTCCCGAG-3'  5'-CggacgttggAATTcGAcggtcaccgaataatccactcaa-3'  5'-gaggtggcggctcgagcGTGCAGATACGGTATTGAGGCAACTC-3'  5'-cggtagggcgaattctaacgTTTacagcaagccatgacttgg-3' |
| --- | --- |

Primers 1–4 were used to amplify the guide strands of the RNAi inverted repeats. Primers 5–6 were used to amplify the *tra-2* intron fragment. Restriction sites are underlined.
